# From Nucleus to No Nucleus: A Multimodal Study of the Toxicity of ZnO Nanoparticles: A Focus on Membrane Integrity, DNA Damage, and Molecular Docking

**DOI:** 10.3390/biology15010023

**Published:** 2025-12-22

**Authors:** Erion Sukaj, Eldores Sula, Ledia Vasjari, Ariol Rama, Erman S. Istifli, Federica Impellitteri, Valbona Aliko, Caterina Faggio

**Affiliations:** 1 Faculty of Medicine and Technical Medical Sciences, Western Balkans University, 1000 Tirana, Albania; erionsukaj@gmail.com (E.S.); eldoressula@info.al (E.S.); 2 Department of Biology, Faculty of Natural Sciences, University of Tirana, 1000 Tirana, Albania; ledia.vasjari@fshn.edu.al (L.V.); ariol.rama@fshn.edu.al (A.R.); 3 NanoAlb, The Albanian NanoScience and Nanotechnology Unit, Academy of Sciences of Albania, 1000 Tirana, Albania; 4 Department of Biology, Faculty of Science and Literature, University of Cukurova, 01250 Adana, Turkey; ermansalih@gmail.com; 5 Department of Veterinary Sciences, University of Messina, 98168 Messina, Italy; federica.impellitteri@studenti.unime.it; 6 Department of Chemical, Biological, Pharmaceutical and Environmental Sciences, University of Messina, 98166 Messina, Italy; cfaggio@unime.it

**Keywords:** zinc oxide nanoparticles, erythrocyte, membrane integrity, nucleated, anucleate, genotoxicity, molecular docking, structural insights

## Abstract

Zinc oxide nanoparticles (ZnO NPs) are increasingly applied in medicine, cosmetics, and environmental technologies, yet their interactions with blood cells remain poorly understood, raising cross-species safety concerns. Using frog (nucleated) and human (anucleate) erythrocytes as comparative models, we show that cellular architecture fundamentally shapes responses to ZnO NPs exposure. Human erythrocytes exhibited a dose-dependent progression from membrane deformation to eryptosis and hemolysis, reflecting the pronounced vulnerability of anucleate cells. In contrast, frog erythrocytes sustained nuclear DNA damage while largely preserving membrane integrity, highlighting the protective or reparative role of the nucleus. Molecular docking revealed energetically favorable interactions of ZnO NPs with ERα-LBD and DNA (ΔG = −4.28 and −5.68 kcal/mol, respectively), while quantum chemical analyses indicated electron-accepting properties and a narrow HOMO–LUMO gap, suggesting efficient macromolecular interactions and intracellular ROS generation. Together, these findings demonstrate that the presence of a nucleus shifts the primary target of nanoparticle toxicity from membrane to genome, providing novel mechanistic insights. This comparative study offers a robust framework for understanding nanomaterial reactivity across taxa and informs One Health-oriented risk assessments.

## Simple Summary

Zinc oxide nanoparticles are tiny particles widely used in medicine, cosmetics, and environmental applications, but their effects on blood cells are not fully understood. In this study, we compared human red blood cells, which lack a nucleus, with frog red blood cells, which have a nucleus, to see how the nucleus affects the response to these nanoparticles. Cells were exposed to different concentrations of zinc oxide nanoparticles, and their health was evaluated by examining changes in cell shape, membrane damage, and DNA integrity. We found that human red blood cells suffered severe membrane damage and died, while frog red blood cells experienced DNA damage, but their membranes stayed mostly intact. Computer simulations showed that zinc oxide nanoparticles can attach to DNA and certain proteins, helping explain how they cause stress inside cells. These results reveal that the presence of a nucleus changes how cells respond to nanoparticles, shifting the main damage from the cell membrane to the DNA. This study provides new insight into why different types of blood cells react differently to nanoparticles and offers valuable information for assessing the safety of these particles for human health and the environment.

## 1. Introduction

Erythrocytes (red blood cells, RBCs) are widely used as a model system to study nanoparticle–cell interactions because of their abundance, simple structure, and well-defined oxidative stress responses. Importantly, vertebrate RBCs differ markedly among taxa: mammalian RBCs are enucleated and lack mitochondria, whereas non-mammalian RBCs (e.g., amphibian or fish erythrocytes) retain nuclei and metabolic activity [1,2,3]. This structural divergence provides a unique toxicological opportunity. Human RBCs, lacking a nucleus, are ideal for probing membrane-level toxicity such as lipid peroxidation and hemolysis [2], while nucleated amphibian or piscine erythrocytes can reveal nuclear and DNA-targeted effects not seen in human blood cells [1,3]. By comparing these two erythrocyte types, one can trace how nanoparticle stress propagates from membrane perturbation to genomic injury—an aspect rarely explored in nanotoxicology.

Zinc oxide nanoparticles (ZnO NPs) are among the most widely used engineered nanomaterials, with applications in cosmetics, pharmaceuticals, food packaging, and environmental remediation [4,5]. However, their extensive use has raised growing concern regarding potential toxicity to human and ecological health. ZnO NPs exert cytotoxic effects primarily through their surface reactivity and the release of Zn^2+^ ions. In biological environments, ZnO dissolution promotes Zn^2+^ release and the generation of reactive oxygen species (ROS) [4,6]. These ROS can oxidize membrane lipids and proteins, impair membrane stability and fluidity, and ultimately lead to hemolysis [7,8]. Meanwhile, excess Zn^2+^ can disrupt ion homeostasis and interfere with enzymatic activity, amplifying oxidative stress and apoptotic signaling [4,9]. Such effects are strongly dependent on nanoparticle characteristics, particularly size and surface area—with smaller ZnO NPs often exhibiting greater reactivity and cytotoxic potential [5,9].

Despite a substantial body of literature documenting ZnO NPs-induced oxidative stress and cytotoxicity in various mammalian cells, the comparative effects across nucleated and anucleated erythrocyte systems remain poorly characterized. Most recent investigations have focused on mammalian systems, embryonic models, or generic in vitro cultures, and several important 2024–2025 studies have reinforced concerns about DNA-damaging potential and hemocompatibility while still leaving cross-taxa comparisons sparse [10,11,12]. Furthermore, while hemolysis and oxidative damage are well-documented endpoints, direct genotoxic evaluation in nucleated erythrocytes—through comet or DNA fragmentation assays—has not been systematically conducted in parallel with membrane-level analyses in human erythrocytes. This gap limits understanding of how structural and metabolic differences between vertebrate erythrocytes influence nanotoxicity mechanisms.

Human erythrocytes, lacking nuclei and mitochondria, respond to ZnO NPs mainly through oxidative and membrane-level processes. Prior studies have reported dose-dependent hemolysis, lipid peroxidation, and eryptosis following ZnO NPs exposure [2,3,8]. In contrast, nucleated erythrocytes—as found in amphibians—retain the capacity to exhibit DNA damage, apoptosis, and genotoxic responses. Recent aquatic and environmental investigations (including Aliko et al., 2024) document ZnO NPs-associated DNA strand breaks and chromatin anomalies in non-mammalian models [13,14]. However, no study to date has conducted a side-by-side comparative assessment linking membrane-level damage in human RBCs with genotoxic outcomes in nucleated erythrocytes under identical exposure conditions. Addressing this gap is critical to elucidate how nanoparticle toxicity shifts from membrane-centered to genome-centered mechanisms depending on cellular architecture.

Computational modeling further enhances mechanistic understanding by simulating how ZnO NPs or Zn^2+^ ions interact with biomolecules. Molecular docking and density functional theory (DFT) analyses have been used to estimate binding affinities and predict interaction sites on proteins and nucleic acids [15,16]. These in silico methods complement experimental results by revealing structure–function relationships at the nanoscale. For instance, Aliko et al. [14] demonstrated that ZnO nanocrystals can stably bind to protein domains, offering insights into redox activity and ion-mediated toxicity. Integrating such computational insights with cellular assays offers a more comprehensive view of ZnO NPs behavior and its potential to induce oxidative and genotoxic stress.

Given the pervasive environmental and biomedical presence of ZnO NPs, a One Health perspective—which recognizes the interconnectedness of human, animal, and environmental health—is essential for nano safety evaluation. Amphibians and fish, as environmental sentinels, provide crucial information about ecological exposure pathways that may parallel human risk [17,18]. Comparative nanotoxicology that spans human and non-mammalian models, therefore, provides translational relevance and helps identify conserved and species-specific mechanisms of nanoparticle-induced damage.

In this context, the present study bridges a major conceptual and methodological gap by performing a comparative evaluation of ZnO NPs toxicity in two evolutionarily distinct erythrocyte systems—nucleated (frog) and anucleated (human). By combining biochemical, oxidative, and genotoxic assays with molecular docking and structural analyses, this work elucidates how the presence of nuclear material modulates the toxicity pathway of ZnO NPs, shifting it from a membrane-focused to a genome-focused process. This integrative approach not only provides a mechanistic basis for species-specific differences in nanoparticle responses but also contributes to the predictive assessment of nanotoxicity across biological systems. Given that recent research (2024–2025) has largely emphasized oxidative or developmental toxicity without exploring the erythrocyte–genotoxic interface, the present study represents a novel and timely contribution to the fields of nano safety and comparative toxicology, offering both conceptual advancement and methodological innovation within a One Health framework.

## 2. Materials and Methods

### 2.1. Study Design and Experimental Overview

This study employed a comparative experimental and computational approach to evaluate the toxicological effects of zinc oxide nanoparticles (ZnO NPs) on human (anucleate) and frog (nucleated) erythrocytes. The workflow integrated in vitro assays to assess oxidative stress, membrane damage, and genotoxicity, with in silico molecular docking and certain quantum chemical analyses to elucidate the intermolecular interactions of ZnO NPs underlying observed cellular responses. Human erythrocytes were used as a model to investigate membrane-level toxicity, including oxidative stress, lipid peroxidation, and hemolysis, while frog erythrocytes were utilized to assess DNA integrity using the comet assay.

### 2.2. Characterization of ZnO Nanoparticles

Zinc oxide nanoparticles (ZnO NPs, <50 nm) were purchased from Sigma-Aldrich Co. GmbH, St. Louis, MO, USA (product code 544906; CAS number: 1314-13-2; white powder; surface area 15–25 m^2^/g; purity > 99%). Morphological and elemental characterization was performed as described in [14], using Scanning Electron Microscopy coupled with Energy Dispersive X-ray Spectroscopy (SEM-EDS; TESCAN VEGA3 LMU, Tescan, Brno, Czech Republic; INCA250 EDS system, Oxford Instruments, Oxford, UK) under high-vacuum conditions (0.018 Pa).

### 2.3. Preparation of ZnO Nanoparticle Suspensions

ZnO NPs were initially suspended in dechlorinated water at 100 mg/L, gently stirred, and sonicated for 30 min using a Q500 Sonicator (Qsonica, Newtown, CT, USA) to ensure homogeneous dispersion. A 100 µg/mL stock solution was prepared by diluting the required mass of ZnO NPs in the sonicated suspension, mixed thoroughly, and stored in a clean, airtight container. Before each experiment, the stock solution was re-sonicated for 15 min. Exposure solutions at 12.5, 25, 50, and 100 µg/mL were prepared by further diluting the stock in the appropriate medium, with a final exposure volume of 1 mL per blood sample.

### 2.4. Human Erythrocyte Preparation

Blood samples from healthy, non-smoking adult volunteers, *n* = 20 (male and female of age 18–24 years old) were collected after written informed consent, in accordance with Albanian Medical Law and Ethics Committee approval (Nr. 10 171, 22/10/09). Volunteers were screened for normal erythrocyte counts and hemoglobin levels. Blood was drawn using a 5 mL syringe with a 22G needle into K_3_-EDTA tubes and processed within one hour. For morphological examination, 1 mL of erythrocytes was separated from plasma by centrifugation (NUVE NF 400, 4000 rpm, 10 min, room temperature) and washed three times with human isotonic solution of 300 mOsm/kg H_2_O. Erythrocytes were then divided into test tubes and incubated with ZnO NPs solutions (12.5, 25, 50, 100 µg/mL) or a control for 3 and 24 h. Morphological changes were observed under a ZEISS Scope. A1 light microscope (Carl Zeiss Microscopy GmbH, Oberkochen, Germany) at 1000× magnification using immersion oil. The plasma-only sample was taken from each tube and examined in a Sysmex XS-1000i Blood Analyzer (Sysmex Corporation, Kobe, Japan) for presence of hemoglobin traces. Methemoglobin (MetHb) levels were determined spectrophotometrically according to Naoum et al. [19]. After exposed to three doses of ZnO NPs, for 3 and 24 h, at 37 °C, the erythrocytes were lysed in hypotonic phosphate buffer (2.5 mM NaH_2_PO_4_, pH 7.4, 4 °C) and centrifuged at 13,000× *g* for 15 min (4 °C), and MetHb concentration was measured against a positive control (no exposed cells to ZnO NPs and treated with 4 mM NaNO_2_ for 1 h at 25 °C).

### 2.5. Amphibian Erythrocyte Preparation

Blood from healthy adult individuals (*n* = 20) of *Pelophylax* spp. was obtained via cardiac puncture using a 2.5 mL syringe with a 23G needle and collected into heparinized tubes. Blood collection followed a minimally invasive procedure conforming to ethical guidelines. Erythrocytes were washed three times and diluted 1:12 (*v*/*v*) in amphibian Ringer’s solution. This nucleated erythrocyte suspension was used for genotoxicity assessment via the comet assay, providing a relevant model for environmental monitoring.

### 2.6. ZnO Nanoparticle Exposure

Human and amphibian erythrocytes were incubated with ZnO NPs solutions (12.5, 25, 50, and 100 µg/mL) for 2, 4, 6, and 24 h at room temperature. Following exposure, samples were collected for DNA damage assessment using the comet assay. All experiments were performed in triplicate.

### 2.7. Oxidative Stress Biomarkers

Oxidative stress in erythrocytes was assessed in accordance with previously described protocols by Aliko et al. [14]. Shortly, malondialdehyde (MDA) as a marker of lipid peroxidation and the activities of antioxidant enzymes catalase (CAT) and superoxide dismutase (SOD) were evaluated according to the methodology explained elsewhere. MDA levels were determined using the thiobarbituric acid-reactive substances (TBARS) assay, while CAT and SOD activities were measured following standard colorimetric protocols. All assays were performed using commercial kits from Sigma-Aldrich (Carlsbad, CA, USA) according to the manufacturer’s instructions. Measurements were carried out in triplicate to ensure reproducibility.

### 2.8. Assessment of DNA Damage Using the Comet Assay

Genotoxic effects in erythrocytes were evaluated using the alkaline Comet assay. Blood samples were embedded in 1% agarose on pre-coated slides, which were dried overnight. For each experimental condition, 20 µL of blood was mixed with 75 µL of 1% agarose on the slide and covered with a coverslip; control samples were treated identically. After the agarose solidified, coverslips were removed, and cells were lysed overnight at 4 °C in lysis buffer. Slides were subsequently rinsed twice with electrophoresis buffer and subjected to electrophoresis at 20 V for 1 h. Following electrophoresis, residual buffer was removed, and DNA was stained with propidium iodide (PI).

Fluorescent images of individual nuclei were captured using an LED fluorescence microscope (Inverted Microscope; IM-5FLD, OPTIKA Microscopes S.r.l., Ponteranica (BG), Italy). DNA damage was quantified using Comet Score 2.0 software, which automatically selects and analyzes comets to reduce observer bias. Key descriptors included comet length, tail length, and the percentage of DNA in the tail (% tail DNA), with the latter used in combination with tail length to calculate the tail moment (Tail DNA% × Tail Length), providing a measure of the extent of DNA damage. For each treatment group, at least 50 randomly selected cells were analyzed to ensure statistical reliability.

### 2.9. Molecular Docking and Structural Analysis

In this context, we selected the estrogen receptor alpha ligand binding domain (ERα-LBD) (PDB ID: 8DUG, resolution: 2.20 Å) (https://www.rcsb.org/structure/8DUG, accessed on 1 November 2025) as the protein receptor to test whether the model ZnO NPs could significantly interact with the ERα-LBD in a manner consistent with ERα-mediated cellular uptake and oxidative stress in human erythrocytes.

To rationalize the experimental DNA damage, on the other hand, observed in frog erythrocytes, we modeled direct ZnO NPs–DNA recognition using the high-resolution canonical B-DNA dodecamer (PDB ID: 355D, resolution: 1.40 Å) (https://www.rcsb.org/structure/355D, accessed on 1 November 2025). This nucleic acid construct provides a well-validated duplex—with defined minor/major grooves and geometry—to assess putative nanoparticle–DNA interactions (groove binding, backbone contacts, nucleobase stacking) that may destabilize the duplex and increase the likelihood of strand breaks and base lesions. Therefore, ERα-LBD was chosen as the receptor consistent with a ZnO NPs–initiated ROS signaling mechanism in human erythrocytes, whereas B-DNA was selected to preliminarily explain the significant nuclear DNA damage observed in frog erythrocytes at the molecular level.

### 2.10. Receptor and Ligand Preparation and Molecular Docking

Before proceeding to docking, missing atoms in the ERα ligand-binding domain (ERα-LBD) and the DNA structures were modeled using Swiss-PdbViewer (v 4.1.0) [20]. To delineate the ERα-LBD active site, we considered the amino acid residues that directly contact the co-crystallized synthetic ERα antagonist (inhibitor T6I-16)—Leu346, Ala350, Glu353, Trp383, Leu387, Met388, Leu391, Arg394, Phe404, Met421, Ile424, Leu525, Val533, and Pro535 in the crystal complex [21,22,23,24,25,26,27,28,29,30,31,32,33,34,35,36,37,38,39,40,41,42,43,44,45,46,47,48,49,50,51,52,53,54,55,56,57,58,59,60,61]. For the B-DNA dodecamer, the entire surface of the 12-nucleotide fragment was designated as the “active site”, since nucleic acids typically lack a single, well-defined binding site and instead present multiple, solvent-exposed pockets [21].

The experimentally observed pristine ZnO unit cell (mp-2133), the nanoparticle model which we also utilized in our previous work [14], was downloaded in .cif format from the Materials Explorer database (https://next-gen.materialsproject.org/materials/mp-2133, accessed on 1 November 2025), subjected to a brief geometry optimization using the Universal Force Field (UFF) and conjugate gradients as the minimization algorithm in Avogadro program to obtain an energetically optimized structure, and exported as a .mol2 file for subsequent docking [22].

In docking experiments of the ZnO NPs with ERα-LBD and double-stranded (ds) DNA, polar hydrogens on the receptors were retained, whereas non-polar hydrogens were merged. Gasteiger charges were then assigned to the ZnO NPs using AutoDockTools 1.5.7 [23], while Kollman charges were applied to ERα-LBD and DNA. The optimized ERα-LBD, DNA, and ZnO NPs structures were subsequently exported in PDBQT format for subsequent docking. Grid boxes were specified as follows: for ERα-LBD, 30 Å × 30 Å × 30 Å (center: x = −7.507 Å, y = −5.286 Å, z = 17.757 Å); for intact DNA, 70 Å × 80 Å × 90 Å points at a spacing of 0.375 Å (center: x = 14.823 Å, y = 21.031 Å, z = 8.577 Å). For DNA, the grid encompassed the entire molecule, thereby covering both major and minor grooves as the search space for ZnO NPs docking. In the grid setup, the boxes were centered at the specified coordinates to allow the ZnO NPs to sample binding pockets on both ERα-LBD and DNA. Docking employed 100 genetic algorithm runs per receptor, with a maximum of an exhaustive 500,000 energy evaluations and 27,000 generations per run. Following docking, AutoDock clustered poses by structural (geometric) similarity (RMSD) and ranked them by the most favorable (lowest) predicted binding energy (ΔG, kcal/mol). The top-ranked ZnO NPs that bind to ERα-LBD and DNA were subsequently analyzed and rendered in Discovery Studio Visualizer v16 (Biovia developed in 2016).

### 2.11. In Silico Quantum Chemical Calculations of ZnO NPs

Frontier-orbital energies (HOMO and LUMO) and the HOMO–LUMO gap were computed from single-point density functional theory (DFT) calculations in PySCF v1 (Python-based Simulations of Chemistry Framework), using the B3LYP exchange–correlation functional and the DGDZVP2 basis set (see https://gaussian.com/basissets/, accessed on 14 December 2025); a combination that has been reported to be relevant in the evaluation of the electronic properties of small ZnO nanoparticles [23,24]. The workflow was adapted from the public script (https://github.com/pritampanda15/Omixium_YouTube_Channel/tree/main/HOMO_LUMO, accessed on 14 December 2025) and slightly modified to read docking poses directly from .sdf files without geometry re-optimization. HOMO–LUMO analyses were performed separately for the ERα-LBD— and B-DNA—docked poses of ZnO NPs, as conformational changes could modulate electronic structure and yield interpretable pose-specific differences [23]. The global reactivity descriptors such as chemical potential (μ), hardness (η), and electronegativity (χ)—were derived using Koopmans theorem: ionization energy (IE) = −E*_HOMO_*, electron affinity (EA) = −E*_LUMO_*; η = (IE − EA)/2, χ = (IE + EA)/2, and μ = −χ.

### 2.12. Statistical Analysis

Data are presented as mean ± standard deviation (SD). Normality of the datasets was assessed using the Shapiro–Wilk test, and homogeneity of variances was verified with Levene’s test. Hemoglobin data were analyzed using two-way ANOVA to evaluate the effects of ZnO-NPs concentration, exposure time, and their interaction, followed by Tukey’s HSD post hoc test when appropriate. Echinocyte data were normally distributed but lacked replication and were, therefore, analyzed descriptively. Relationships between hemoglobin levels and echinocyte counts were assessed using Pearson’s correlation. Statistical significance was set at *p* < 0.05. All analyses and figure generation were performed using GraphPad Prism 9.0 (GraphPad Software, San Diego, CA, USA) and R version 4.3.3 (R Core Team, Vienna, Austria, 2025).

## 3. Results

### 3.1. ZnO NPs Characterization

**Figure 1:**
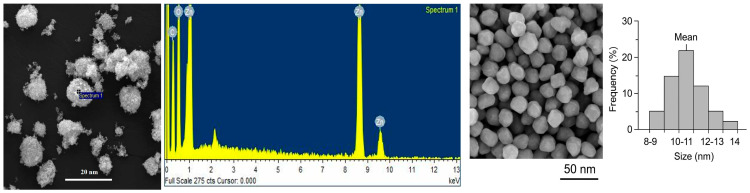
Characterization and size distribution of ZnO NPs and SEM-EDS spectra.

According to the SEM-EDS analysis, using a TESCAN VEGA3 LMU (Tescan, Brno, Czech Republic) scanning electron microscope, equipped with an EDS INCA Energy 250XT system (Oxford Instruments, Oxford, UK), revealed that ZnO NPs had a spherical shape with a size ranging from 8 to 15 nm in diameter, Figure 1. The samples analyzed were mounted on the sample holder with double-sided adhesive conductive carbon tape, in high vacuum mode (pressure of 0.018 Pa). The spectrum obtained through EDX characterization of the sample confirmed the purity of ZnO NPs.

### 3.2. Morphological Results in 3 and, 24 h Exposure of Erythrocytes to ZnO NPs

Starting from previous comparative studies reporting that lower-vertebrate nucleated erythrocytes have lower antioxidant enzyme levels than human cells but greater membrane stability [25], reflecting distinct antioxidative defense mechanisms, we designed our experiments to apply biomarker endpoints appropriate to each erythrocyte model and to use oxidative stress enzymes as the common comparative point.

**Figure 2:**
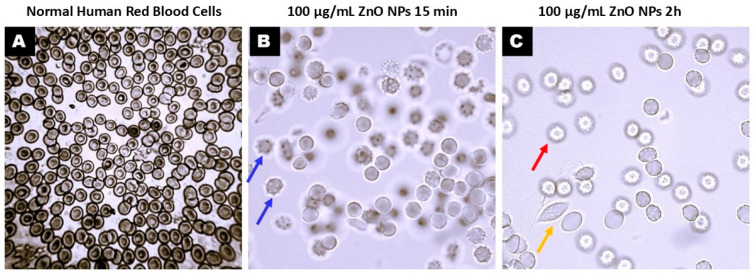
Human erythrocyte membrane alterations. Light microscopy images showing (**A**) normal human erythrocytes, (**B**,**C**) erythrocyte with surface blebs (echinocytes and acanthocytes, blue arrows; leptocytes, yellow arrows; and ghost cells, red arrows). Human erythrocytes were untreated (**A**) or treated with 100 µg/mL ZnO nanoparticles for 15 min and 2 h at 37 °C magnification 1000×.

Under light microscopy examination the presence of echinocytes (abnormal erythrocytes) was detected in each concentration following first 3 h of exposure, when the massive red blood cell membrane alterations happened, Figure 2.

Light microscopy analysis revealed notable morphological transformations in human erythrocytes following exposure to ZnO nanoparticles (ZnO NPs) (Figure 2). Untreated cells displayed the typical biconcave disc shape with smooth membranes (Figure 2A). In contrast, ZnO NPs-treated erythrocytes exhibited prominent membrane irregularities, including surface blebbing and the appearance of echinocytes, acanthocytes, leptocytes, and ghost cells (Figure 2B,C). Quantitative assessment of echinocyte formation, performed by manual counting in ten randomly selected high-power fields (1000× magnification, oil immersion), demonstrated a clear concentration-dependent trend. The proportion of echinocytes increased from less than 5% at the lowest ZnO NPs concentration to more than 50% at the highest, relative to the total erythrocyte population. Statistical analysis confirmed a significant positive correlation between ZnO NPs concentration and echinocyte frequency (Pearson’s r = 0.956; *p* = 0.044, significant at α = 0.05), indicating a direct relationship between nanoparticle dose and membrane deformation severity

### 3.3. Hemolysis and Echinocytosis Results for 3 and 24 h of Exposure to ZnO NPs

**Figure 3:**
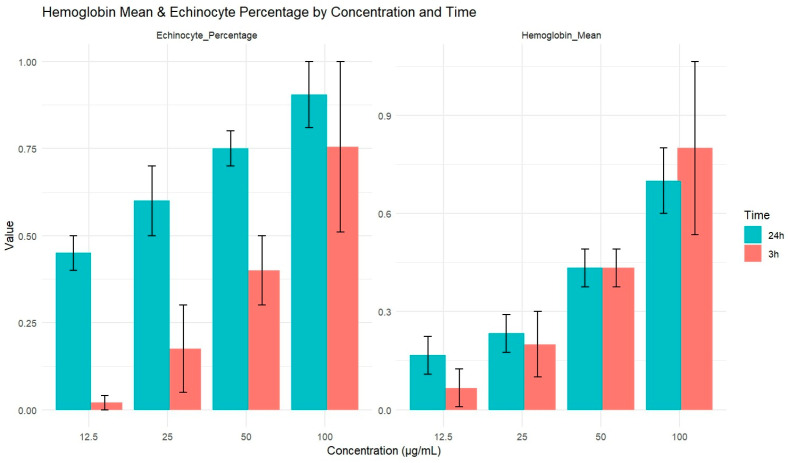
Comparison graphics of hemolysis (Hemoglobin concentration values) and mean echinocytes number in different ZnO NPs concentrations for both 3 and 24 h exposure.

The presence of hemoglobin in plasma was assessed following exposure to varying concentrations of ZnO NPs at two points: 3 h and 24 h. For each concentration, the analysis was performed in triplicate. A dose-dependent increase in hemoglobin levels was observed for both exposure durations. The hemoglobin values obtained after 3 h, and 24 h of exposure are presented in Figure 3.

**Figure 4:**
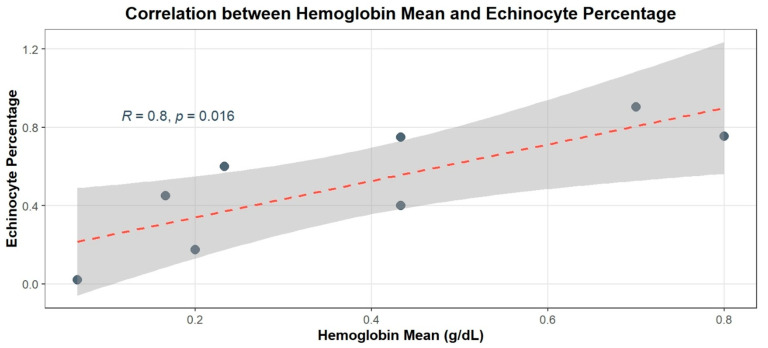
Correlation between hemolysis rate (hemoglobin mean) and echinocyte formation (cell deformity).

Statistical analysis revealed that the concentration of ZnO NPs had a significant effect on hemolysis (F = 35.365, *p* < 0.001), while neither time nor the interaction between time and concentration had a significant effect. Specifically, at 3 h, the Pearson correlation coefficient between ZnO NPs concentration and hemoglobin levels was 0.997 (*p* = 0.003), and at 24 h, the correlation remained high with a coefficient of 0.995 (*p* = 0.005), confirming a significant and sustained dose-dependent relationship over time. Additionally, Pearson/Spearman correlation analysis was conducted to explore the relationship between hemoglobin levels and echinocyte formation. The results revealed a strong positive correlation (r = 0.803, *p* = 0.016), suggesting a linear relationship between these two variables, Figure 4.

### 3.4. Antioxidant Enzyme Activities and Lipid Peroxidation Marker

**Figure 5:**
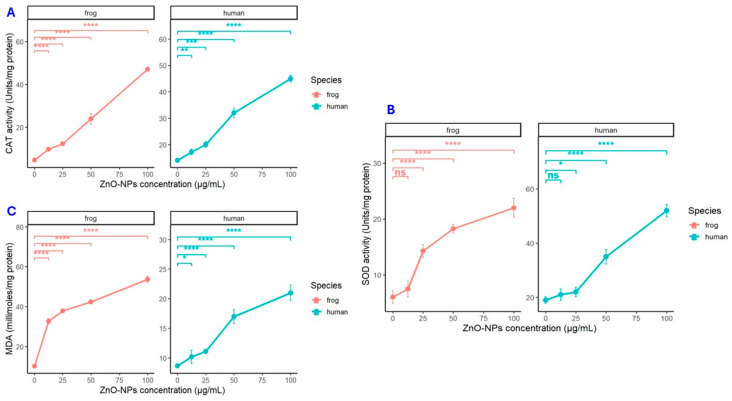
Effects of ZnO NPs on antioxidant enzyme activities and lipid peroxidation. (**A**) Catalase (CAT) activity, (**B**) Superoxide dismutase (SOD) activity, and (**C**) Malondialdehyde (MDA) levels in human and frog samples exposed to 0 (Control), 12.5, 25, 50, and 100 µg/mL of ZnO NPs. Data represent mean ± SD (*n* = 10), * *p* < 0.01, ** *p* < 0.001, *** *p* < 0.0001, **** *p* < 0.00001 vs. control; *ns* for non-significant (one-way ANOVA followed by Tukey’s post hoc test).

The activities of catalase (CAT) and superoxide dismutase (SOD) were measured in human and frog samples exposed to increasing concentrations (12.5–100 µg/mL) of the tested compound (Figure 5A,B, Table 1). CAT activity exhibited a concentration-dependent increase in both species. In humans, CAT values rose significantly from 12.5 µg/mL onward (*p* < 0.05) and peaked at 100 µg/mL (** *p* < 0.001). A similar pattern was observed in frogs, with a moderate increase at 25 µg/mL (*p* < 0.05) and a pronounced enhancement at 50–100 µg/mL (* *p* < 0.01, ** *p* < 0.001).

SOD activity followed the same concentration-dependent trend. In human samples, SOD increased from 19 ± 1.2 U/mg protein (control) to 52 ± 2.3 U/mg protein at 100 µg/mL, with significance achieved at concentrations above 25 µg/mL (* *p* < 0.01). Frog SOD activity increased from 6.02 ± 1.12 to 22 ± 1.7 U/mg protein, indicating a progressive and statistically significant rise (*p* < 0.05 at 25 µg/mL; * *p* < 0.01 and ** *p* < 0.001 at higher concentrations).

Malondialdehyde (MDA) levels, a biomarker of lipid peroxidation, also increased in both human and frog samples in a dose-dependent manner (Figure 5C, Table 1). In humans, MDA levels rose significantly from 25 µg/mL onward (* *p* < 0.01), whereas frog tissues exhibited a sharper response, with elevated MDA detected as early as 12.5 µg/mL (*p* < 0.05) and reaching maximal values at 100 µg/mL (** *p* < 0.001). These results collectively indicate that the treatment induces oxidative stress, stimulating the upregulation of antioxidant enzymes (CAT and SOD) alongside increased lipid peroxidation, with frogs displaying a greater sensitivity to the treatment compared to human samples.

### 3.5. DNA Damage Assessment

**Figure 6:**
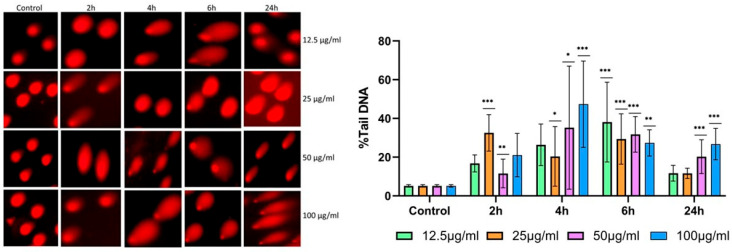
Fluorescence images obtained from single-cell alkaline gel electrophoresis (Comet Assay) of control and exposed frog erythrocytes 2, 4, and 6 h after exposure to increasing doses of ZnO NPs (12.5, 25, 50 and 100 µg/mL). DNA was stained with propidium iodide (PI). Fluorescence images were captured using an LED fluorescence microscope (Seria IMP-5 FLD). For each sample, 50 comets were randomly selected and analyzed with Comet Score 2.0 software. Values are expressed as the mean ± standard error of the mean (SEM). Statistical analysis was performed using an unpaired *t*-test. * *p* < 0.05, ** *p* < 0.001, *** *p* < 0.0001 (*p* < 0.05 was considered statistically significant).

Following a 24 h exposure of frog erythrocytes to zinc oxide nanoparticles (ZnO NPs) at four concentrations (12.5, 25, 50, and 100 µg/mL), DNA damage increased progressively with nanoparticle concentration (Figure 6). Specifically, both comet length and tail length were longer compared with the untreated control, indicating that ZnO NPs induced DNA strand breaks and compromised genomic integrity in a dose-dependent manner.

### 3.6. Molecular Docking

**Figure 7:**
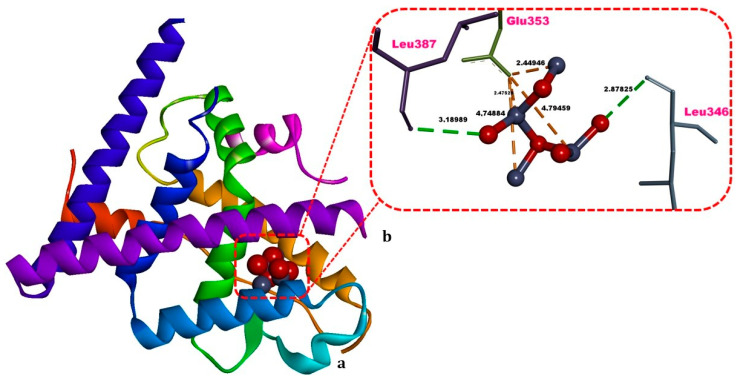
Structure of the zinc oxide nanoparticle (ZnO NPs) in complex with the estrogen receptor alpha ligand-binding domain (ERα-LBD). (**a**) Three-dimensional overview of the ERα-LBD–ZnO NPs complex; (**b**) zoomed-in view of the binding site highlighting interfacial contacts. Hydrogen bonds (H-bonds) are indicated by green dashed lines, and electrostatic interactions are depicted with orange dashed lines. Non-bonded contact distances (Å) are labeled in bold black. Images were visualized and rendered using Discovery Studio v16.

ZnO NPs displayed an energetically favorable binding energy (ΔG = −4.28 kcal/mol) in the ligand binding domain of estrogen receptor alpha (ERα-LBD). The ligand–receptor interactions comprised hydrogen (H) bonds with Leu346 and Leu387, numerous electrostatic interactions with Glu353, together with many van Waals contacts involving Leu349, Ala350, Leu391, Arg394, Leu403, Phe404 (Table 2, Figure 7).

**Figure 8:**
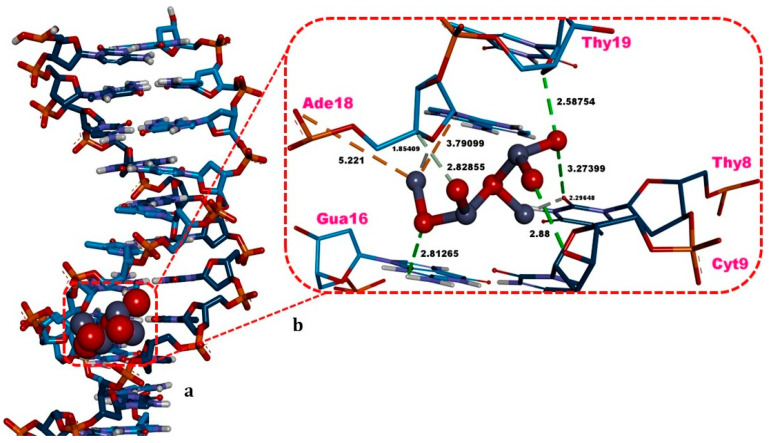
Structure of the zinc oxide nanoparticle (ZnO NPs) in complex with the double-stranded B-DNA dodecamer. (**a**) Three-dimensional overview of the DNA–ZnO NPs complex; (**b**) zoomed-in view of the DNA binding mode of ZnO NPs, highlighting interfacial contacts. Hydrogen bonds (H-bonds) and carbon–hydrogen (C–H) bonds are indicated by green and light-green dashed lines; metal–acceptor interactions are shown with grey dashed lines; and electrostatic interactions are depicted with orange dashed lines. Non-bonded contact distances (Å) are labeled in bold black. Images were visualized and rendered using Discovery Studio v16.

ZnO NPs exhibited an energetically more favorable binding energy (ΔG = −5.68 kcal/mol) against the B-DNA dodecamer displaying a minor groove binding mode. The complex of ZnO NPs against the double-stranded DNA receptor showed H-bonds with Thy8, Cyt9, Gua16, Ade18, Thy19, electrostatic interactions with Ade18, van der Waals contacts with Thy8, Gua10, Ade17, Thy19, and metal-acceptor interactions with Thy8 (Table 2, Figure 8).

The docking binding energy values calculated for ERα-LBD (as well as for the DNA receptor) indicate that these binding reactions are thermodynamically favorable (exergonic) and may occur spontaneously; however, we regard these docking scores as primarily hypothesis-generating, and a more accurate assessment of the true binding strength would require endpoint free energy calculations, such as MM-GBSA or MM-PBSA.

For the two docked poses of the ZnO NPs against ERα-LBD and DNA, the HOMO and LUMO energies, the HOMO–LUMO energy gap, chemical potential, hardness, and electronegativity are summarized in Table 3.

Table 3 summarizes the HOMO/LUMO energies, HOMO–LUMO gap, and conceptual descriptors (μ, η, χ) for ZnO NPs docked to ERα-LBD and B-DNA. For the ERα-LBD–bound pose of ZnO NPs, the frontier orbitals were found as E*_HOMO_* = −6.47 eV and E*_LUMO_* = −5.45 eV, yielding a narrow 1.02 eV gap; the associated descriptors are μ = −5.96 eV, η = 0.51 eV, and χ = 5.96 eV. In the B-DNA–bound pose of ZnO NPs, the frontier levels shift slightly lower (E*_HOMO_* = −6.60 eV; E*_LUMO_* = −5.71 eV), tightening the gap to 0.89 eV and giving μ = −6.16 eV, η = 0.44 eV, and χ = 6.16 eV—a marginally softer and more electronegative state. Using Koopmans-type relations, both poses display electron-accepting character (χ > 0), with the B-DNA pose slightly softer (lower η) and more electronegative (higher χ) than the ERα-LBD–bound pose.

Thus, while differences are modest, the B-DNA–bound conformation of ZnO NPs is predicted to be marginally more reactive and more prone to accept electron density, consistent with an increased propensity for charge-transfer interactions at the DNA interface, which could accompany non-covalent contacts as observed in the docking. Overall, the quantum chemical descriptors qualitatively support the view that ZnO NPs docked against ERα-LBD and DNA could act as an electron acceptor in the cellular milieu and may participate in redox processes (e.g., ROS-related interactions), which could indirectly increase the membrane and DNA damage observed in human and frog erythrocytes, respectively.

## 4. Discussion

Our dual-cell erythrocyte model provides novel insights into the differential effects of zinc oxide nanoparticles (ZnO NPs) on cells with distinct structural and functional characteristics [1]. It is important to note that ZnO NPs toxicity can arise from the particles themselves, their aggregates, or released Zn^2+^ ions. Although SEM characterization (8–15 nm) was performed, aggregation, surface charge, and ion dissolution in biological media were not assessed. Therefore, the observed effects should be interpreted as resulting from “*ZnO nanoparticles and/or their dissolved Zn^2+^ ions*”, transparently acknowledging this limitation while maintaining the validity of the comparative findings.

By comparing nucleated amphibian erythrocytes with anucleate human erythrocytes, we were able to discriminate between membrane-targeted and nuclear-level effects of ZnO NPs. This comparative strategy surpasses conventional single-species assays, offering a broader perspective on nanoparticle behavior across biologically diverse systems. Human erythrocytes, lacking nuclei and mitochondria, serve as a physiologically relevant model for evaluating oxidative and hemolytic responses reflective of human exposure [4,8]. In contrast, nucleated amphibian erythrocytes retain metabolic activity and genomic material, enabling the detection of genotoxic effects and highlighting interactions with DNA and nuclear structures [24]. The distinct responses observed between these two erythrocytes types underscore how cellular complexity modulates the pathways of nanoparticle-induced toxicity, shifting the damage focus from the membrane toward genomic targets [10].

Previous studies, particularly those involving human or other anucleate red blood cells, have rarely examined membrane destabilization (e.g., hemolysis or morphological alterations) and genetic damage (e.g., comet assay or micronuclei formation) in combination under nanoparticle exposure. Most reports have focused on a single endpoint, often limited to oxidative or morphological effects. For instance, work on human erythrocytes exposed to ZnO nanoparticles demonstrated pronounced hemolysis linked to oxidative stress and lipid peroxidation but did not extend to direct genotoxicity evaluation [25,26,27]. Although significant progress has been made in exploring ZnO NP toxicity across diverse cell types, including studies reporting hemolysis and ROS generation in erythrocytes [28]—mechanistic investigations specifically targeting human erythrocytes remain limited.

The present study contributes to this gap by providing direct experimental evidence of concentration-dependent morphological and biochemical alterations in human erythrocytes following ZnO NPs exposure. We observed a clear, dose-related increase in echinocyte formation, which increased progressively with NP concentration, from fewer than 5% at low doses to over 50% at high doses, indicating strong nanoparticle–membrane interactions [29,30].

Hemolysis followed a similar pattern, reinforcing the hypothesis that structural and compositional features of erythrocytes determine their susceptibility to ZnO NPs-induced damage. The two-way ANOVA showed that ZnO NPs concentration had a highly significant effect on hemoglobin release (F = 35.365, *p* < 0.001), whereas exposure time and the concentration × time interaction were not significant. Although the experiment included both short-term (3 h) and long-term (24 h) exposures following standard hemolysis protocols, the lack of a time effect indicates that membrane damage in human erythrocytes occurs rapidly and reaches its maximal level within the first 3 h. This aligns with the strong dose-dependency observed at both time points and suggests that ZnO NPs-induced hemolysis is rapid rather than progressive over prolonged exposure. Strong positive correlations between ZnO NPs concentration and hemoglobin levels at both 3 h (r = 0.997, *p* = 0.003) and 24 h (r = 0.995, *p* = 0.005) supported a robust, sustained hemolytic response. Strong correlations between echinocyte formation and hemolysis (r = 0.803, *p* = 0.016) and between NP concentration and hemoglobin release at both 3 h (r = 0.997, *p* = 0.003) and 24 h (r = 0.995, *p* = 0.005) indicate a mechanistic link between early membrane deformation and subsequent cell lysis. The strong but not perfect correlation between echinocytosis and hemolysis (r = 0.803) is expected, as echinocyte formation represents an early and partly reversible membrane response that does not always progress to lysis. In parallel, hemolysis may also occur through shape-independent pathways, including oxidative lipid damage or direct nanoparticle–membrane interactions [6,29,30].

Mechanistically, the transition of erythrocytes into echinocytes reflects early disruption of lipid bilayer organization, ATP depletion, or oxidative modification of membrane components, while hemolysis represents terminal membrane rupture [6,12,14]. The ability of ZnO NPs to elicit both responses at environmentally relevant concentrations raises significant concerns regarding hemocompatibility under chronic or repeated exposure. These results support membrane destabilization as a primary mechanism of ZnO NPs toxicity in human erythrocytes, mediated by nanoparticle surface reactivity and oxidative stress [6].

Our findings are consistent with prior reports in human erythrocytes, where ZnO NPs induced dose-dependent hemolysis via ROS generation and lipid peroxidation, although DNA strand breakage has not been documented in these anucleate cells [25,31,32]. Extending this paradigm, our data demonstrate that exposure to increasing ZnO NPs concentrations triggers a pronounced oxidative stress response in both human and frog erythrocytes, as indicated by the dose-dependent elevation in SOD and CAT activities accompanied by increased MDA levels (Figure 5, Table 1).

In human erythrocytes, the increase in antioxidant enzyme activity likely reflects a compensatory response to heightened ROS, which correlates with the observed hemolysis and the elevated incidence of echinocyte formation. These morphological alterations suggest that excessive oxidative stress compromises membrane integrity, triggering both structural deformation and cell lysis [33,34,35]. The more marked elevation of MDA in frog erythrocytes, coupled with results from the comet assay, indicates substantial oxidative DNA damage, highlighting a heightened susceptibility of amphibian cells to lipid peroxidation and genomic instability under similar conditions [13,14,24]. The observed species-specific responses suggest distinct pathways by which oxidative stress impacts erythrocyte integrity.

While CAT and SOD activity and MDA levels indicate an oxidative stress response in both human and frog erythrocytes, direct measurement of intracellular ROS was not performed in this study. Including such measurements in future work would help confirm the mechanistic origin of the oxidative damage and further clarify species-specific differences in the cellular response to ZnO NPs exposure.

Formation of echinocytes and increased hemolysis in human cells suggests that lipid peroxidation and ROS disrupt membrane phospholipid asymmetry, leading to shape changes and premature cell rupture [35]. Indeed, the high polyunsaturated fatty acid content of erythrocyte membranes renders them particularly vulnerable to oxidative damage and hemolysis [30]. In this study, the upregulation of SOD and CAT in human erythrocytes likely reflects an adaptive attempt to counter elevated ROS levels; however, beyond a threshold, antioxidant defenses appear insufficient to prevent structural compromise. In frog erythrocytes, the substantial rise in MDA and positive comet assay results point to oxidative stress-driven DNA fragmentation rather than over membrane lysis, consistent with observations of nucleated erythrocytes undergoing genotoxic damage prior to hemolysis in aquatic species [36,37,38,39,40].

Human RBCs incubated with ZnO NPs (<50 nm) exhibited substantial hemoglobin release and morphological transformations—including echinocyte formation—correlated with oxidative stress markers [29]. This divergence may reflect inherent differences in membrane composition, antioxidant capacity, or DNA packaging between species. Together, these findings highlight that oxidative stress can simultaneously drive morphological alterations, hemolysis, and genotoxicity, but the predominant damage pathway can vary by species [41,42,43,44]. Understanding these mechanistic nuances provides valuable insight into evaluating hematological and genomic risks of oxidative agents across vertebrate models, with potential implications for environmental toxicology and pharmacological safety assessment. Collectively, these findings underscore the interplay between oxidative stress, membrane fragility, and genotoxicity, emphasizing that both species exhibit dose-dependent cellular stress responses, albeit with distinct vulnerabilities: membrane destabilization predominates in human erythrocytes, whereas oxidative DNA damage is more pronounced in frog erythrocytes.

Earlier studies on NP-exposed erythrocytes have shown that membrane anchoring and NP-mediated lipid damage led to spiculated echinocyte forms and subsequent hemolysis, reinforcing our observation of a strong positive correlation between echinocyte percentage and hemoglobin release. Thus, our results corroborate these findings while extending them by quantifying morphological (echinocytosis) and functional (hemolysis) endpoints simultaneously. This strengthens the mechanistic link between membrane deformation and erythrocyte lysis in the context of ZnO NPs exposure [29,30]. Importantly, our study introduces a novel quantitative perspective on erythrocyte shape transformation as an early and sensitive biomarker of NP-induced toxicity. Unlike hemolysis, which reflects terminal membrane rupture, echinocytosis indicates sublethal damage and membrane remodeling, thereby offering valuable insight into early NP–cell interactions [13,14,24,34].

In nucleated amphibian erythrocytes, ZnO NPs exposure induced both membrane alterations and genotoxic effects. Comet assay analysis revealed increased tail DNA percentages and longer tail moments, indicative of DNA strand breaks [13,14,24]. The alkaline comet assay used in this study detects DNA strand breaks but does not distinguish specific lesion types, such as oxidative modifications like 8-OHdG. While our oxidative stress data suggest that ROS-mediated damage likely contributes to the observed genotoxicity in frog erythrocytes, the exact nature of the DNA lesions remains to be determined. Future studies using oxidative lesion-specific assays would help clarify the precise mechanisms of ZnO NPs-induced DNA damage.

Elevated SOD and CAT activities corroborated a ROS-driven stress response. These findings align with previous observations in aquatic vertebrates, where nucleated erythrocytes exhibit DNA fragmentation and oxidative lesions under NP exposure [36,37,38,39,40,45,46,47]. Thus, while oxidative stress compromises membranes in human erythrocytes, it affects both nuclear and membrane integrity in amphibian cells, reflecting species-specific vulnerabilities.

Oxidative stress emerged as a central mediator in both cell types. In human erythrocytes, increased antioxidant enzyme activity represents a compensatory response to ROS, with lipid peroxidation compromising membrane integrity and triggering echinocytosis and hemolysis [34,35,43]. In amphibian erythrocytes, the pronounced rise in malondialdehyde and positive comet assay results indicate that oxidative stress predominantly drives DNA damage rather than hemolysis [13,14,24]. Additionally, released Zn^2+^ ions may interact with membrane-bound enzymes or redox-sensitive proteins, further amplifying oxidative stress and membrane distortion [47].

The interspecies differences observed in our study are consistent with previously reported evidence that erythrocyte stability varies widely across vertebrates due to species-specific antioxidant defense mechanisms. Comparative analyses have shown that nucleated erythrocytes from lower vertebrates generally possess lower baseline levels of antioxidant enzymes compared to mammalian erythrocytes, yet paradoxically exhibit greater resistance to membrane oxidative damage, indicating a more robust structural organization and different stress-response strategies [47,48]. This phenomenon has been attributed to the distinct antioxidative architecture and metabolic demands of nucleated red cells, which can tolerate oxidative challenges without undergoing rapid membrane disruption [41,42,43,44]. In line with these findings, the oxidative stress biomarkers measured in our study (CAT, SOD, MDA), Figure 5, provide a quantitative basis for interspecies comparison: human erythrocytes displayed sharp increases in membrane-associated oxidative damage, whereas frog erythrocytes, despite their lower antioxidant enzyme capacity, maintained membrane integrity but showed genotoxic alterations. These differences justify the inclusion of oxidative enzyme assays in both models, as oxidative stress represents the common pathway through which the two erythrocyte types can be directly compared despite their fundamentally different structural and physiological responses to ZnO NPs exposure.

Our results demonstrate that zinc oxide nanoparticles induce pronounced oxidative stress and lipid peroxidation in human erythrocytes. These findings indicate that ZnO NPs compromise erythrocyte membrane integrity and cytoskeletal architecture, consistent with mechanisms of nanoparticle-induced oxidative/nitrosative stress reported in the literature [6,49,50,51]. Human erythrocytes, despite being anucleate, express functional membrane-associated estrogen receptors (ERα and ERβ) capable of initiating rapid, nongenomic signaling through kinase pathways such as ERK1/2 and AKT [3,48,49]. Although we did not directly measure receptor activation or nitric oxide production, the observed oxidative and structural alterations are consistent with the downstream consequences of receptor-mediated signaling pathways reported in other studies.

To gain molecular-level insights into ZnO NPs–induced membrane injury in human erythrocytes and DNA damage in frog erythrocytes, we complemented our experimental observations with molecular docking and density functional theory (DFT) analyses to elucidate structure–activity relationships (SAR). The ZnO NPs demonstrated a thermodynamically favorable binding affinity (ΔG = –4.28 kcal/mol) toward the ligand-binding domain of human estrogen receptor-α (ERα-LBD) (Table 2, Figure 7). Human erythrocytes express functional ERα and ERβ, and membrane-associated (nongenomic) ERα signaling triggered by xenobiotic interactions can phosphorylate ERK1/2 and AKT and increase nitric oxide (NO) production. Dysregulated ERα activation and excess NO are known to mediate peroxynitrite-driven alterations in cell morphology and cytoskeletal architecture [48,49,50,51].

In line with these mechanisms, membrane accumulation of ZnO NPs may engage ERα-mediated nongenomic uptake and signaling, promoting oxidative/nitrosative stress and lipid peroxidation, thereby compromising erythrocyte membrane integrity and triggering hemolysis [6]. Experimentally, we observed a dose-dependent progression from echinocytosis → eryptosis → hemolysis in human erythrocytes, accompanied by marked oxidative stress, whereas nucleated frog erythrocytes displayed pronounced nuclear DNA damage despite relatively stable membranes. Previous studies on plastic particulates in erythrocytes have shown plasma-membrane binding and internalization co-localized with ERα/ERβ, coupled with activation of non-genomic ER signaling (pERK1/2, pAKT) and robust ROS generation, ultimately perturbing membrane homeostasis [33,49].

Furthermore, subsets of ERα and ERβ have been shown to translocate to the plasma membrane, where they can bind diverse small molecules [48]. These findings could support ERα binding as a plausible molecular-initiating event for erythrocyte membrane internalization of nanoparticles and, therefore, motivated our use of ERα-LBD as a structurally well-characterized possible molecular target of ZnO NPs, without implying that it is the sole or predominant in vivo target for ZnO NPs. Thus, we propose that ZnO NPs, acting as putative ligands, may exploit increased cytosol-to-membrane trafficking and abnormal ER distribution in erythrocytes to engage ERα, facilitating receptor binding, internalization, and downstream oxidative effects.

Our experimental data showed a dose-dependent transition from echinocytosis → eryptosis → hemolysis with marked oxidative stress in human erythrocytes, whereas frog erythrocytes (nucleated) exhibited significant nuclear DNA damage despite comparatively more stable membranes. Prior work on plastic particulates in erythrocytes demonstrates plasma-membrane binding and internalization that co-localize with estrogen receptor (ER) α/ERβ, accompanied by activation of non-genomic ER signaling (pERK1/2, pAKT) and robust ROS generation, ultimately perturbing membrane homeostasis [49,50]. These observations support Erα-binding as a plausible molecular-initiating event for NP–erythrocyte interactions and, moreover, a subset of ERα—as well as ERβ—has been shown translocate to the plasma membrane, where they bind diverse small molecules [48]. Thus, we hypothesized that the increased cytosol-to-plasma-membrane trafficking and the abnormal distribution of ERs in erythrocytes could be consistent with a model in which ZnO NPs, acting as putative ligands, engage ERα to drive receptor binding and internalization.

Docking study using the dsDNA dodecamer demonstrated that ZnO NPs also exhibit an energetically favorable affinity (ΔG = −5.68 kcal/mol) toward this biomolecule, adopt a minor groove binding mode, and exhibit an interaction profile biased toward Ade and Thy (Table 2, Figure 8). An experimental study reported that ZnO NPs bind to calf thymus (CT) DNA in an entropy-driven manner and that this binding is energetically favorable (Δ*G* = −5.07 kcal/mol) using the isothermal titration calorimetry, which is close to the value observed in our docking (Δ*G* = −5.68 kcal/mol) [51,52,53,54]. When considered alongside the docking results, this experimental finding supports the view that the substantial DNA damage observed in our study in frog nucleated erythrocytes following 24 h exposure and interval-based ZnO NPs treatment (2, 4, and 6 h) could arise from the direct interaction of ZnO NPs with DNA and the subsequent induction of DNA damage [14,24,34,52,53].

In the quantum chemical analysis, it was found that both ERα-LBD– and B-DNA–bound poses of the ZnO NPs displayed frontier-orbital and global reactivity descriptors consistent with an electron-accepting character (Table 2); a physicochemical profile that provides a plausible basis for cellular electron uptake and subsequent intracellular ROS generation. It should be emphasized, however, that the ZnO unit cell model used in this study represents a simplified approximation of the experimental 8–15 nm nanoparticles and does not explicitly include surface defects, hydration, or protein/DNA-induced conformational changes; accordingly, the reported frontier orbital energies and conceptual DFT descriptors should be viewed as qualitative indicators of the relative electron-accepting character at the ERα-LBD versus DNA interface, rather than as precise HOMO–LUMO energy gaps for the actual nanoparticles. Concordantly, in avian (chicken) red blood cells (RBCs), ZnO NPs increased ROS levels, induced hemolysis, and produced significant DNA damage in the comet assay under 24 h exposure [6,29], demonstrating that ZnO NPs–erythrocyte interactions can directly translate into oxidative membrane injury and nuclear lesions in erythrocytes. Moreover, in mammalian erythrocyte and lymphocyte cells, ZnO NPs triggered ROS-mediated toxicity, including lipid peroxidation (LPO) of cellular membranes and oxidative DNA damage that results in genomic injury and cell death [55,56,57,58,59,60,61].

Taken together, combining experimental findings with literature evidence, molecular docking, and quantum chemical analyses, ZnO nanoparticles (ZnO NPs) appear to exert a dual mode of toxicity in erythrocytes: ROS-mediated oxidative stress compromising membrane integrity, and potential direct interactions with ERα or DNA that may underlie genotoxic effects. These mechanisms highlight the importance of assessing both nucleated and anucleate erythrocytes to capture the full spectrum of nanoparticle-induced damage.

## 5. Conclusions

This study offers the first integrative comparison of ZnO NPs toxicity in nucleated versus anucleate erythrocytes, revealing complementary but mechanistically distinct responses. Human erythrocytes exhibited dose-dependent echinocytosis and hemolysis linked to oxidative membrane injury, whereas nucleated frog erythrocytes displayed both membrane destabilization and DNA strand breaks. Docking studies against ERα-LBD and DNA, together with quantum chemical descriptors, support a model in which ZnO NPs simultaneously trigger oxidative membrane damage and engage receptor- or DNA-mediated pathways. This dual-cell approach establishes a versatile framework for nano safety assessment, demonstrating that models limited to anucleate cells may underestimate the full biological impact of nanoparticles on nucleated systems.

## Figures and Tables

**Table 1:** One-way ANOVA summary for the effects of compound concentrations on oxidative stress biomarkers (SOD, CAT, and MDA) in human and frog erythrocytes (Note: **** *p* < 0.0001).

Parameter	Species	Source of Variation	df	F-Value	*p*-Value	Significance
SOD (U/mg protein)	Human	Between groups	4	152.6	<0.0001	****
		Within groups	10	-	-	-
	Frog	Between groups	4	178.3	<0.0001	****
		Within groups	10	-	-	-
CAT (U/mg protein)	Human	Between groups	4	426.5	<0.0001	****
		Within groups	10	-	-	-
	Frog	Between groups	4	615.2	<0.0001	****
		Within groups	10	-	-	-
\MDA (mmol/mg protein)	Human	Between groups	4	240.1	<0.0001	****
		Within groups	10	-	-	-
	Frog	Between groups	4	692.7	<0.0001	****
		Within groups	10	-	-	-

**Table 2:** Docking-predicted binding poses, binding affinities, and residue interaction patterns for the zinc oxide nanoparticle (ZnO NPs) with the estrogen receptor-α ligand binding domain (ERα-LBD) and a B-DNA dodecamer (12-mer).

Ligand	Receptor	Binding Mode	Binding Energy * (ΔG: kcal/mol)	H-Bond	Electrostatic (Attractive, Pi-cation)	Van der Waals	Other (Metal-Acceptor)
ZnO NPs	ERα-LBD	—	−4.28	Leu346 (2.87 Å), Leu387 (3.18 Å)	Glu353 (2.44 Å, 2.47 Å, 4.74 Å, 4.79 Å)	Leu349, Ala350, Leu391, Arg394, Leu403, Phe404	—
ZnO NPs	B-DNA	Minor groove	−5.68	Thy8 (3.27 Å), Cyt9 (2.88 Å), Gua16 (2.81 Å), Ade18 (2.82 Å), Thy19 (2.58 Å)	Ade18 (3.79 Å, 5.22 Å)	Thy8, Gua10, Ade17, Thy19	Thy8 (2.29 Å), Ade18 (1.85 Å)

* Binding energy (kcal/mol): The predicted change in free energy upon formation of the ligand–target complex; more negative values indicate stronger predicted binding affinity. The reported values in the table correspond to the top-ranked conformations of ZnO NPs with the most negative binding energy.

**Table 3:** Predicted quantum chemical descriptors for two docked conformations of the ZnO NPs.

Docked Conformation	E*_HOMO_*	E*_LUMO_*	Energy Gap	μ	η	χ
ERα-LBD bound pose	−6.47	−5.45	1.02	−5.96	0.51	5.96
B-DNA bound pose	−6.60	−5.71	0.89	−6.16	0.44	6.16

ERα-LBD: Estrogen receptor alpha ligand binding domain (PDB ID: 8DUG). B-DNA: Canonical B-DNA dodecamer (PDB ID: 355D). μ: Chemical potential. η: Hardness. χ: Electronegativity.

## Data Availability

The data supporting the findings of this study are available from the corresponding author upon reasonable request.

## Abbreviations

The following abbreviations are used in this manuscript:

ZnO NPs	Zinc Oxide Nanoparticles
CAT	Catalase
SOD	Superoxide dismutase
MDI	Malondialdehyde
